# Forced expression of IL-7R promotes CD8 T cell cytotoxicity to self antigen

**DOI:** 10.1371/journal.pone.0188112

**Published:** 2017-12-22

**Authors:** YuFeng Peng

**Affiliations:** Division of Rheumatology, Department of Medicine, University of Washington, Seattle, Washington, United States of America; La Jolla Institute for Allergy and Immunology, UNITED STATES

## Abstract

Cross-presentation of apoptotic cell associated antigens by immature dendritic cells prevents the activation of self reactive CD8 T cells. Tolerized self reactive CD8 T cells down-regulate IL-7R expression on their surface. Whether over-expression of IL-7R can reverse their fate and function has not been examined. In this paper, we showed forced expression of IL-7R in OT-I T cells by a transgene enhanced CD8 T cell mediated diabetes in the RIP-mOVA model. Although IL-7R Tg (transgenic) did not completely reverse the deletion of OT-I T cells, it provided a significant survival advantage over w.t OT-I T cells. Furthermore, IL7R Tg OT-I T cells isolated from diabetic pancreata displayed increased production of IFN-γ, higher expression of T-bet, and increased externalization of CD107a. We also found that immature DCs containing apoptotic cells expressed high levels of PDL-1 on their surface. Although IL-7R Tg did not change PD1 expression on activated OT-I cells *in vivo*, the transgene enabled a significantly lower number of OT-I T cells to induce diabetes in the absence of PDL-1. Our results demonstrated that forced expression of IL-7R not only improved the functionality of tolerized CD8 T cells, it also acted in synergy with PDL-1 deficiency to further promote CD8 T cell cytotoxicity to self antigens.

## Introduction

Apoptotic cells are an important source of self antigens. Most apoptotic cell associated antigens are phagocytosed and presented by immature dendritic cells. However, unlike mature dendritic cells, immature dendritic cells lack the requisite costimulatory molecules and cytokines to induce CD8 T cell immunity. CD8 T cells activated by immature DCs are functionally impaired, they neither produce IFN-γ nor kill targets efficiently [1]. Moreover, immature DCs have a longer life span than mature DCs *in vivo* [2]. Chronic presentation of self antigen can lead to CD8 T cell exhaustion. In combination, these unique features of immature DCs contribute to the tolerization of self reactive CD8 T cells *in vivo*. Once tolerized, self reactive CD8 T cells become refractory to restimulation *in vitro* (anergy) and some of them are deleted. Both surviving anergic and deleted CD8 T cells down regulate IL-7R expression [3].

It is well established that the IL-7/IL-7R pathway is required for T cell survival [4]. IL-7R deficient mice and humans lack mature T cells in the periphery [5]. Over-expression of Bcl-2 restored the T cell population in IL-7 deficient mice [6]. Conversely, IL-7 up-regulates important survival factors such as Bcl-2 and Mcl-1 in T cells [7]. *In vivo*, IL-7 is primarily expressed by stromal cells [8], and its level is mainly regulated by consumption. Under conditions of lymphopenia, the lack of T cell competition raises the level of IL-7 in serum [9]. The IL-7R consists of the IL-7R α chain and a common gamma chain. Both IL-7 and TCR can modify the expression level of the IL-7R α chain on T cells. Whereas the down-regulation of IL-7R through IL-7 is transient, the loss of IL-7R driven by TCR is more prolonged [10].

The balance between TCR and IL-7R signals determines the fate of naïve CD8 T cells; therefore, continuous signaling through the IL-7R is not always beneficial to their survival. Kimura et al. have shown that forced expression of IL-7R led to IFN-γ induced cell death of CD8 T cells [11]. Homeostatic interactions between the TCRs and self antigens blocks IL-7R signaling and rescues naive CD8 T cells from IL-7 induced cell death. Whether antigen specific interactions between autoreactive TCRs and self antigens would have the same effect remains to be determined.

In both mice and humans, recombinant IL-7 has been demonstrated to dramatically affect T cell expansion [4]. T cells with the highest expression of IL-7R, including recent thymic emigrants and the naive subset, are the most sensitive. Recombinant IL-7 also has an adjuvant effect on T effector cells. In a mouse model of tumor vaccination, recombinant IL-7 boosted CD4 T cell function and overcame the inhibitory network imposed by the suppressive tumor microenvironment [12]. Conversely, in mouse models of multiple sclerosis and type I diabetes, antibody mediated blocking of IL-7R signaling preferentially inhibited the expansion of Th17 and Th1 cells, respectively [13–15].

Recent studies have linked IL-7 signaling with the PDL-1/PD-1 pathway [13, 15]. As a critical co-inhibitory molecule for CD8 T cell function [16], PDL-1 is highly expressed by subsets of myeloid cells and dendritic cells *in vivo*; some of them are specialized in the uptake and processing of apoptotic cells [17]. PD-1, the receptor of PDL-1, is expressed by activated CD4 and CD8 T cells. Both PDL-1 deficient and PD1 deficient mice developed autoimmune diseases [18, 19]. More importantly, PDL-1 deficiency not only restored the function of exhausted viral specific CD8 T cells during chronic viral infection but also reversed T cell anergy in autoimmunity [20, 21]. Modification of PD-1 expression by exogenous IL7 and anti-IL7R antibody can partially explain their effects on T cell functions. For example, IL-7 treatment of T cells *in vitro* down-regulated their PD1 expression [13, 15], while antibody mediated blocking of IL-7R *in vivo* increased the frequency of PD1+ T cells [15].

Although exogenous IL-7 can overcome immune tolerance, it remains to be established whether over-expression of IL-7R will have a similar effect. In this paper, we examined whether forced expression of IL-7R will restore the function of tolerized CD8 T cells and whether the revived CD8 T cells will co-operate with PDL-1 deficiency to further enhance their cytotoxicity to self antigen.

## Materials and methods

### Mice and antibodies

RIP-mOVA, RIP-sOVA, and actin-mOVA mice were purchased from Jackson Laboratory. OT-I mice and Vβ5x RIP-mOVA mice were kindly provided by Dr. Mike Bevan (University of Washington). hCD2-IL7R Tg mice were provided by Dr. Keith Elkon (originally from Dr. A. Singer, NIH). PDL-1 deficient mice were from Dr. Latchman (University of Washington). All Mice were backcrossed onto the C56BL/7 background for more than 10 generations and used in the study at 8–12 week of age. All animal (mice) usage and procedures in this study were approved by the IACUC of the University of Washington. All mice were euthanized with CO_2_. The number of animals used in each study was estimated using the following parameters: number of tails = 1, effect size = 2, α = 0.05, and power = 0.8. Antibodies against IL-7R (A7R34), CD45.1 (A20), CD45.2 (104), IFN-γ XMG1.2), and CD8α (53.67) were obtained from Biolegend. Antibodies against T-bet (4B10), PD-1 (RMP1-30), PDL-1(MIH-5), PDL-2 (TY25), B7-1 (16-10A1), B7-2 (GL1), and B7H-2 (MIH12) were from eBioscience.

### Preparation of apoptotic cell loaded dendritic cells (DCs)

Apoptotic cells were loaded with OVA or BSA by osmotic shock as previous described [22]. Immature DCs were derived from bone marrow in the presence of 10 ng/ml murine GM-CSF (Peprotech) for 6–7 days. Apoptotic cells were mixed with immature DCs at a 5:1 ratio for 12 hours. To activate DCs, immature DCs loaded with Apo-OVA (apoptotic cell-OVA) were stimulated with LPS (1μg/ml) for 6 hours. In phagocytosis assays, apoptotic cells were labeled with PKH67 green dye (Sigma), as previously described [22]. DCs with phagocytosed apoptotic cells were identified by flow cytometry as CD11c+PKH+ cells.

### Isolation of OT-I T cells

OT-I T cells from spleens were purified by negative selection using a CD8 T cell isolation kit from Miltenyi. More than 80% of the transferred cells were CD8+. In some experiments, total spleen T cells were purified by a pan T cell isolation kit from Miltenyi.

### Adoptive transfer and subsequent cell isolation from recipients and gene expression analysis

2 x 10^6^ CD45.1+ OT-I T cells isolated by negative selection were transferred into CD45.2+ B6 hosts by i.v.. One day later, 2 x10^6^ CD45.2+ bone marrow derived DCs containing apoptotic cells were transferred to the recipients. Seven days later, spleen cells from the 5 recipients were harvested and pooled. OT-1 T cells were purified using biotinylated anti-CD45.1 and streptavidin microbeads (Miltenyi). The purity of recovered cells was 70–80%, and host cell contamination was less than 10%. RNA from purified cells was isolated using Trizol (Invitrogen) and reverse transcribed to cDNA using the SuperScript III kit (Invitrogen). Gene expression was analyzed by real time PCR using SYBR® Green PCR Master Mix (Applied Biosystems). The primers were synthesized by Integrated DNA Technologies; their sequences are listed in Table 1.

**Table 1:** Primers for real time PCR.

Gene	Forward Primer (5'->3')	Reverse Primer (5'->3')
Emoes	ACCAATAACAAAGGTGCAAACAAC	TGGTATTTGTGCAGAGACTGCAA
T-bet	GTTCCCATTCCTGTCCTTC	CCTTGTTGTTGGTGAGCTT
Perforin	TTTCGCCTGGTACAAAAACC	CGTTCAGGCAGTCTCCTACC
Granzyme B	ACTCTTGACGCTGGGACCTA	AGTGGGGCTTGACTTCATGT
IFN-γ	GAGGAACTGGCAAAAGGATG	TGAGCTCATTGAATGCTTGG
Ly6c	GCAGTGCTACGAGTGCTATGG	ACTGACGGGTCTTTAGTTTCCTT
NKG2D	GAAACAGGATCTCCCTTC	CCGAGACAATTCTCTTG
IL-7R	GCGGACGATCACTCCTTCTG	AGCCCCACATATTTGAAATTCCA
CD122	GAAGTGCTCGACGGAGATTC	GAAGTAGCCCTGGTTGGTGA
Bcl-2	CTGCACCTGACGCCCTTCACC	CACATGACCCCACCGAACTCAAAGA
Bcl-x	GATCCCCATGGCAGCAGTAAAGCAAG	CCCCATCCCGGAAGAGTTCATTCACT
CD8α	GCTCAGTCATCAGCAACTCG	ATCACAGGCGAAGTCCAATC

### Diabetes and insulitis assessment

Different numbers of w.t and IL-7R Tg OT-I T cells were transferred into RIP-mOVA mice via intravenous injection. Diabetes was monitored every other day by urine glucose testing using Diastix (Bayer). Mice with two consecutive glucose readings of 4+ (approximate 55 mmol/L urine glucose) were considered diabetic [23]. Diabetes was confirmed by measuring blood glucose level (>18mmol/ml) using test strips and the Contour monitoring system (Bayer). Whole pancreas (including head, body, and tail) was fixed in formalin and embedded in paraffin blocks. Five 5-μm thick sections (30μm apart) were cut from each block and stained with H&E. Briefly, deparaffinized sections were re-hydrated and stained with Mayer’s Hematoxylin Solution for 2 minutes. After rinsing with warm running tap water (15 minutes), slides were counter-stained with Eosin Y Solution for 2 minutes. The slides were then dehydrated, cleared, and mounted with resinous mounting medium. Five sections per pancreas and an average of 10 islets per mouse were scored blindly for insulitis. The area of lymphocytic infiltration in each islet was further quantified by Image J (intact islets = no infiltrate, peri-insulitis = 0–30%, insulitis = > 30%).

### Isolation of lymphocytes from islets and intracellular staining

Infiltrating lymphocytes in islets were isolated as described [24]. Briefly, pancreatic tissues were collected and digested with Liberase RI (Roche Applied Science) at 37°C for 15–20 min. Dissociated islets were isolated under a dissecting microscope. To release intra-islet lymphocytes, islets were digested with Trypsin-EDTA for 10 min, followed by treatment with cell dissociation buffer (GIBCO/BRL) for 15 min at 37°C. After overnight incubation at 37°C, 4-5x10^4^ cells (70–80% viable) were recovered from pooled pancreata from 5 mice. Lymphocytes were then fixed and externalization of CD107a (1D4B, Biolegend) was determined by flow cytometry. To detect IFN-γ production, lymphocytes were re-stimulated with 1μM OVA peptide (SIINFEKL) in the presence of Golgi blocker (BDbiosciences) for 5 hours. Cells were fixed and permeabilized according to the manufacturer's instructions. The level of intracellular IFN-γ was detected by APC labeled anti-IFN-γ (XMG1.2, Biolegend). To detect T-bet and pStat5, cells were fixed using BD Phosflow Lyse/Fix Buffer and stained with PE labeled anti-T-bet (4B10, eBioscience) and anti-pSTAT5 (pY649, BDbiosciences), respectively.

### ELISA

Nunc maxisorp plates were coated with 2μg/ml capture antibody diluted in PBS (clone JES6-1A12 for IL2, clone AN-18 for IFN-γ) overnight at 4°C. After blocking with PBS/1% BSA, the plates were incubated with neat supernatant samples overnight at 4°C. Cytokine levels were detected using biotinylated antibodies (clone JES6-5H4 for IL2, clone XMG1.2 for IFN-γ) diluted in PBS/1%BSA, followed by HRP conjugated streptavidin. Recombinant IL2 and IFN-γ were used as standards. All reagents were purchased from Biolegend.

### *In vitro* stimulation of T cells with apoptotic cells

Thymocytes from actin-mOVA mice were isolated and exposed to 60 mJ /cm^2^ UV light by a cross-linker. 90% of the cells became apoptotic (Annexin V + PI+) after 2 hours. W.t. and IL-7R Tg OT-I T cells were purified from spleens, and stimulated with apoptotic thymocytes in the presence or absence of 10ng/ml recombinant murine IL-7 (Peprotech). Supernatant samples were collected at day 3 and the levels of IL-2 and IFN-γ were evaluated by ELISA.

### Analysis of T cell anergy

5x10^6^ purified OT-I T cells were transferred into RIP-mOVA mice via intravenous injection. At day 7 after transfer, total lymphocytes from pancreatic draining LNs (lymph nodes) (anergized) and distal draining LNs (naive) were re-stimulated with OVA peptide for 3 days *in vitro*. T cell proliferation was assayed by adding H^3^ thymidine to the culture for the final 14 hours. The proliferation index was calculated as: H^3^ counts of stimulated T cells/ H^3^ counts of un-stimulated T cells. The levels of IL-2 and IFN-γ in the supernatant were assessed by ELISA.

### Statistical analysis

Log-rank tests were used for diabetes incidences, one tailed Mann-Whitney U tests were used for insulitis percentages, and two tailed student t tests were used in all other experiments. P values less than 0.05 were considered significant. * p<0.05, ** p<0.01, *** p < 0.001.

## Results

### Cross-presentation of apoptotic cell associated antigen down-regulates IL-7R on CD8 T cells

**Fig 1:**
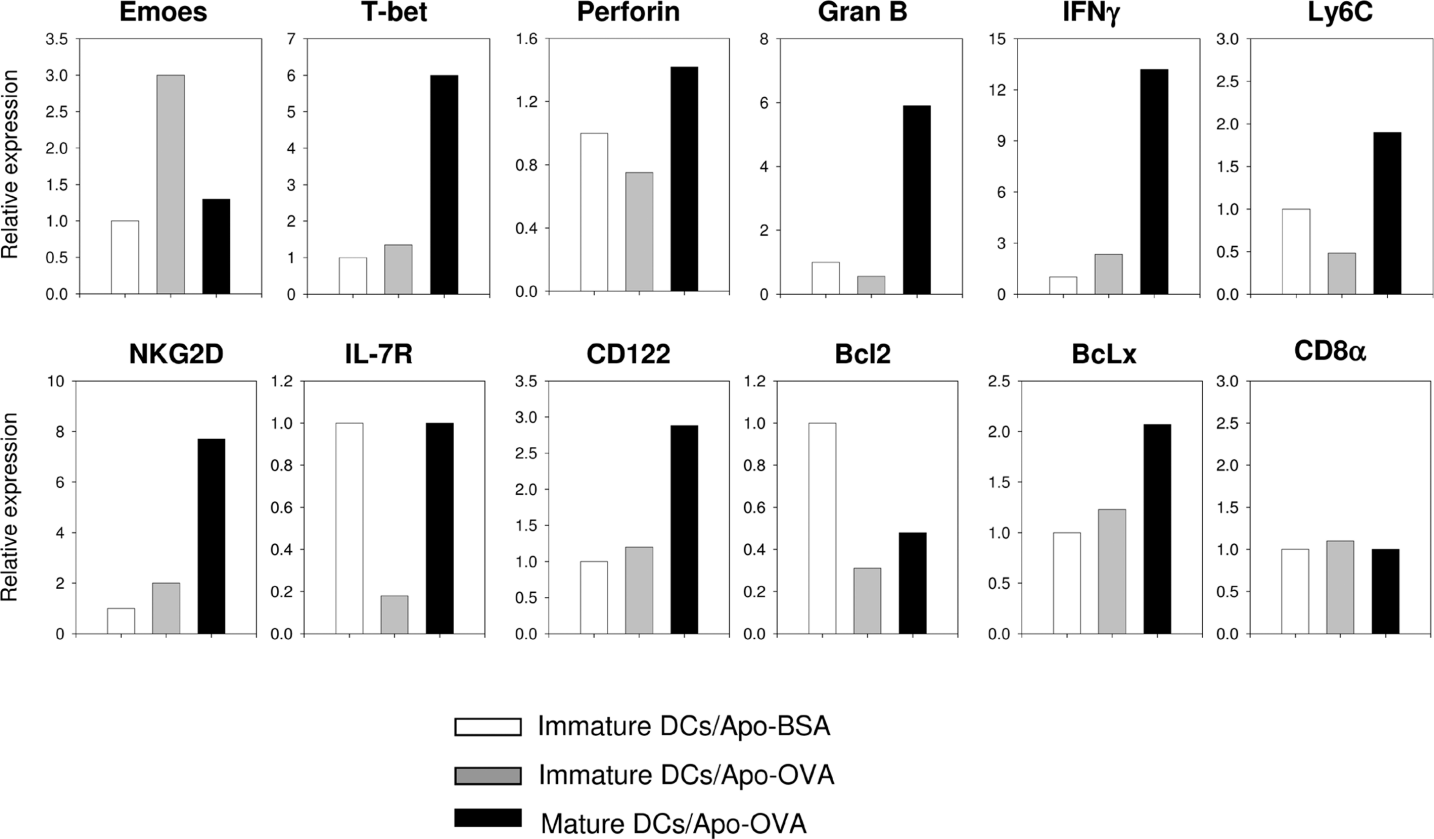
Cross-presentation of apoptotic cell associated antigen by immature dendritic cells down-regulates IL-7R on CD8 T cells. 2 x10^6^ purified CD45.1+ OT-I T cells were transferred into CD45.2+ mice. One day after the transfer, recipients were challenged with 2 x 10^6^ immature dendritic cells loaded with Apo-BSA, or 2 x 10^6^ immature dendritic cells loaded with Apo-OVA, or 2 x 10^6^ LPS matured dendritic cells loaded with Apo-OVA (n = 5 for each group). At day 7 after challenge, spleen cells from each group were pooled and the transferred OT-I T cells were recovered using anti-CD45.1 biotin beads. RNA from the pooled samples was isolated and reverse-transcribed to cDNA. Relative levels of mRNAs of indicated genes were determined by real-time PCR. Similar results were obtained from two experiments.

Many cell types have the potential to induce T cell tolerance. To determine whether antigen presentation by immature dendritic cells alone is sufficient to induce the phenotype of tolerized CD8 T cells, we first transferred CD45.1+ OT-I CD8 T cells into CD45.2+ mice. We then challenged the recipients with immature DCs loaded with Apo-BSA, immature DCs loaded with Apo-OVA, or LPS stimulated DCs preloaded with Apo-OVA. At day 7 after challenge, we isolated OT-I cells and compared the expressions of genes critical for the function and survival of CD8 T cells. As expected, OT-I T cells stimulated without antigen remained naïve, while those challenged with mature DCs acquired the phenotypes of effector CD8 T cells (including high expression of T-bet, Granzyme B, and IFN-γ). On the contrary, OT-I T cells stimulated with immature DCs loaded with APO-OVA showed a small increase of IFN-γ and acquired the Emoes high/T-bet low phenotype typical of terminally exhausted CD8 T cells found during chronic viral infection [25, 26]. Moreover, expression of IL-7R on the tolerized CD8 T cells was markedly lower than both naïve and effector T cells (**Fig 1**), suggesting constant antigen presentation by immature DCs might limit the function and the survival of CD8 T cells through IL-7R down-regulation.

**Fig 2:**
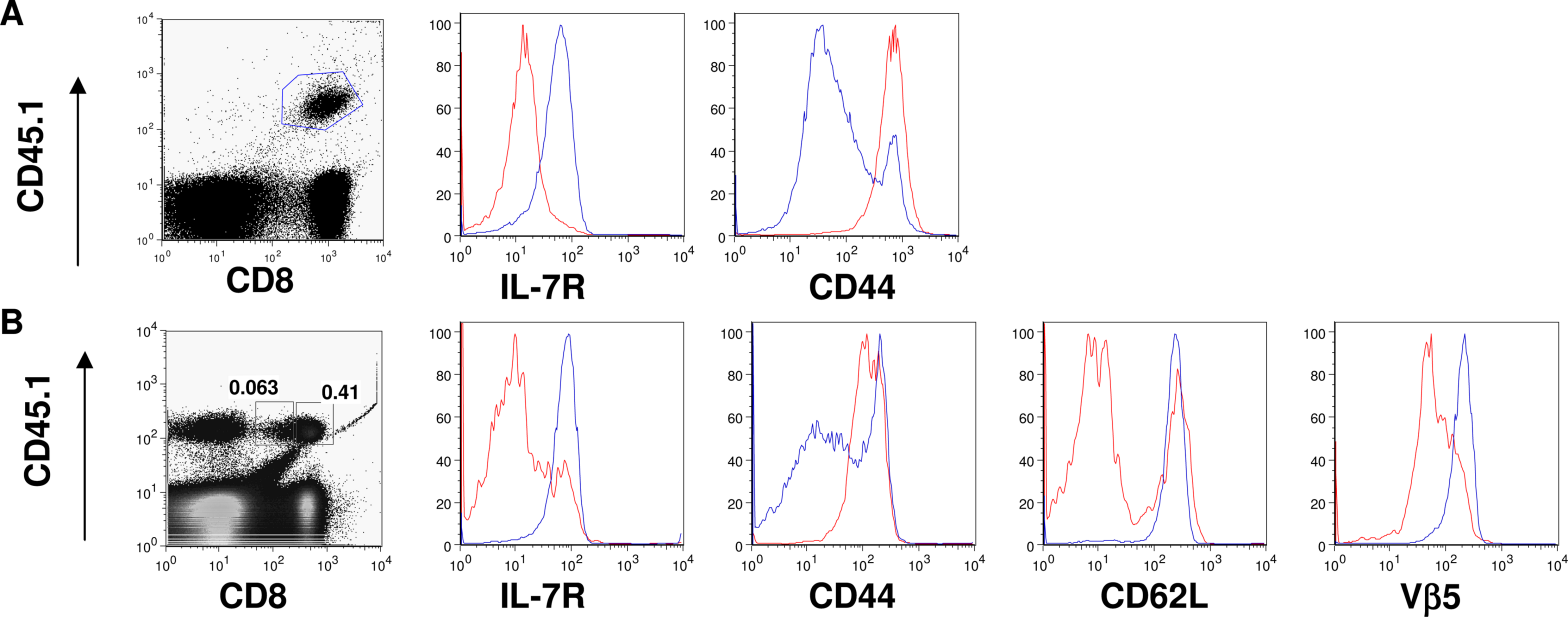
Cross-presentation of endogenous self antigen by immature DCs down-regulates IL-7R expression on auto-reactive CD8 T cells. **A**: 2x 10^6^ purified CD45.1+ OT-I T cells were transferred into CD45.2+ RIP-mOVA mice (n = 5). At day 7 after transfer, lymphocytes were recovered from the pancreatic draining LNs and distal LNs. OT-I T cells were identified by flow cytometry using CD45.1 marker and their expression of IL-7R and CD44 were determined (pancreatic LNs-red line, distal LNs-blue line). The results are representative of at least 3 independent experiments. **B**: 2x 10^6^ CD45.1+ total spleen T cells from Vβ5xRIP-mOVA mice were transferred into K14-mOVA mice (n = 5). At day 7 after transfer, lymphocytes were recovered from the skin draining LNs and transferred T cells were identified by CD45.1. Autoreactive and non-autoreactive CD8 T cells were distinguished by their level of CD8 expression (CD8 low- autoreactive, CD8 high- non-autoreactive) and their expression of IL-7R, CD44, CD62L, and Vβ5 were determined using flow cytometry (CD8 low–red, CD8 high-blue). Similar results were obtained from two independent experiments.

Next, we investigated whether cross-presentation of an endogenous antigen would lead to a similar down-regulation of IL-7R on CD8 T cells. To this end, we transferred OT-I T cells into RIP-mOVA mice. In RIP-mOVA mice, membrane bound OVA is expressed specifically in the β cells of the pancreas and the OVA antigen is cross-presented by immature DCs in the pancreatic draining LNs but not in the distal LNs [27]. As shown in **Fig 2A**, at day 7 post transfer, OT-I T cells recovered from pancreatic draining LNs had significantly lower expression of IL-7R than those from distal LNs.

The TCR expressed by OT-I T cells has a high affinity to OVA antigen [28]. However, under normal condition, CD8 T cells with high affinity to self antigens are deleted in the thymus; only those with low affinity circulate in the periphery. This selection of self reactive T cells was modeled in the Vβ5x RIP-mOVA mice [29]. In the Vβ5xRIP-mOVA mice, while CD8 T cells with high affinity to OVA antigen were deleted in the thymus, low affinity CD8 T cells escaped to the periphery. These surviving self reactive CD8 T cells can be distinguished from non self reactive T cells by their decreased cell surface expression of CD8 and Vβ5 [29]. To determine whether cross-presentation of endogenous self antigen can down regulate IL-7R on low affinity self-reactive CD8 cells, we transferred splenic T cells from Vβ5xRIP-mOVA mice into K14-mOVA mice in which membrane bound OVA is specifically expressed in keratinocytes [30]. As shown in **Fig 2B**, in K-mOVA recipients, CD8 low Vβ5 low self reactive T cells exhibited the phenotype of activated T cells (CD44 high, CD62L low) and their surface expression of IL-7R was concomitantly down-regulated. Therefore, in the presence of cross-presentation of self antigen, both low and high affinity self reactive CD8 T cells down-regulate their IL-7R expression.

### Forced expression of IL-7R enhances OT-I T cell mediated diabetes

**Fig 3:**
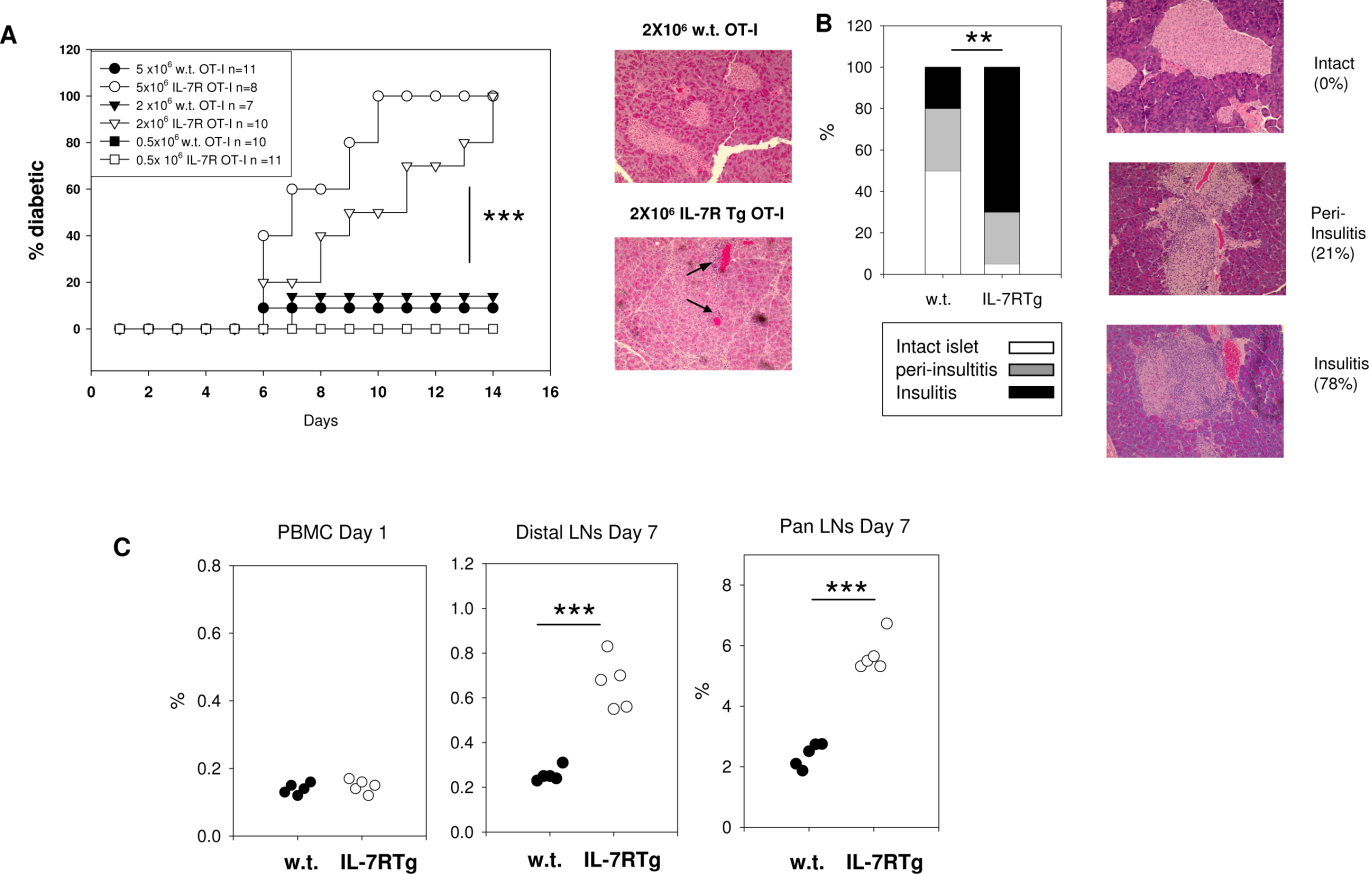
Forced expression of IL-7R enhances OT-I T cell mediated diabetes. **A:** Various numbers (5x 10^6^, 2 x 10^6^, 0.5 x10^6^) of w.t. (closed symbol) or IL-7R Tg (open symbol) OT-I T cells were transferred into RIP-mOVA mice (n = 7–11 in each group). Left: Diabetes incidence in the recipients. IL-7R Tg vs. w.t in 5 x 10^6^ group: p<0.001; IL-7R Tg vs. w.t in 2 x 10^6^ group: p<0.001 (log-rank test). Right: Representative H&E staining of pancreata from mice that have received 2 x 10^6^ OT-I cells. Note: in mice injected with IL-7R Tg OT-I T cells, most islets in the pancreas were destroyed (arrows). **B:** Left: summary of insulitis. 5 sections per pancreas and an average 10 islets per mouse were scored blindly for insulitis (n = 5 in each group). Right: representative H&E staining of pancreata used in insulitis scoring. The percentage of infiltrated area in the islet was quantified by Image J: intact islet = no infiltration, peri-insulitis = 0–30%, insulitis = 30%- 90%. **C:** 2 x10^6^ purified OT-I T cells from w.t. or IL-7R Tg mice were transferred into RIP-mOVA mice (n = 5). The percentages of OT-I T cells recovered from the recipients are shown (left: PBMC (peripheral blood mononuclear cell) at day1, middle: distal LNs at day7, right: pancreatic draining LNs at day7). Total numbers of lymphocytes recovered from two groups and their viabilities were similar (distal LN: w.t (1.2 +/- 0.1 x 10^6^) vs. IL7R Tg (1.4 +/-0.2 x 10^6,^ >90% viable), pancreatic draining LN: w.t. (1.2+/- 0.3 x 10^6^) vs. IL7R Tg (1.3+/-0.1 x 10^6,^ >90% viable).

After transfer into RIP-mOVA mice, OT-I T cells undergo a transient activation followed by anergy and deletion [31]. Since IL-7/IL-7R signaling regulates the survival and the functionality of T cells [4], we aimed to determine whether over-expression of IL-7R could overcome the impaired function of tolerized OT-I T cells. To this end, we first crossed OT-I TCR transgenic mice with hCD2-IL7R transgenic mice (IL-7R Tg) [32]. We then transferred different numbers of w.t. or IL-7RTg OT-I T cells into RIP-mOVA mice. As shown in **Fig 3A**, both 5x10^6^ and 2x 10^6^, but not 0.5x10^6^, IL-7R Tg OT-I T cells were able to induce earlier onset and higher frequency of diabetes in RIP-mOVA mice than the same numbers of w.t. OT-I T cells. Histological analysis of the pancreata confirmed the destruction of islets and extensive infiltration of lymphocytes (**Fig 3B**). Furthermore, we recovered 3-fold more IL-7R Tg OT-I cells than w.t OT-I cells from the LNs of the recipient RIP-mOVA mice at day 7 after the transfer (**Fig 3C**).

### Forced expression of IL-7R enhances the survival of OT-I T cells in RIP-mOVA mice

**Fig 4:**
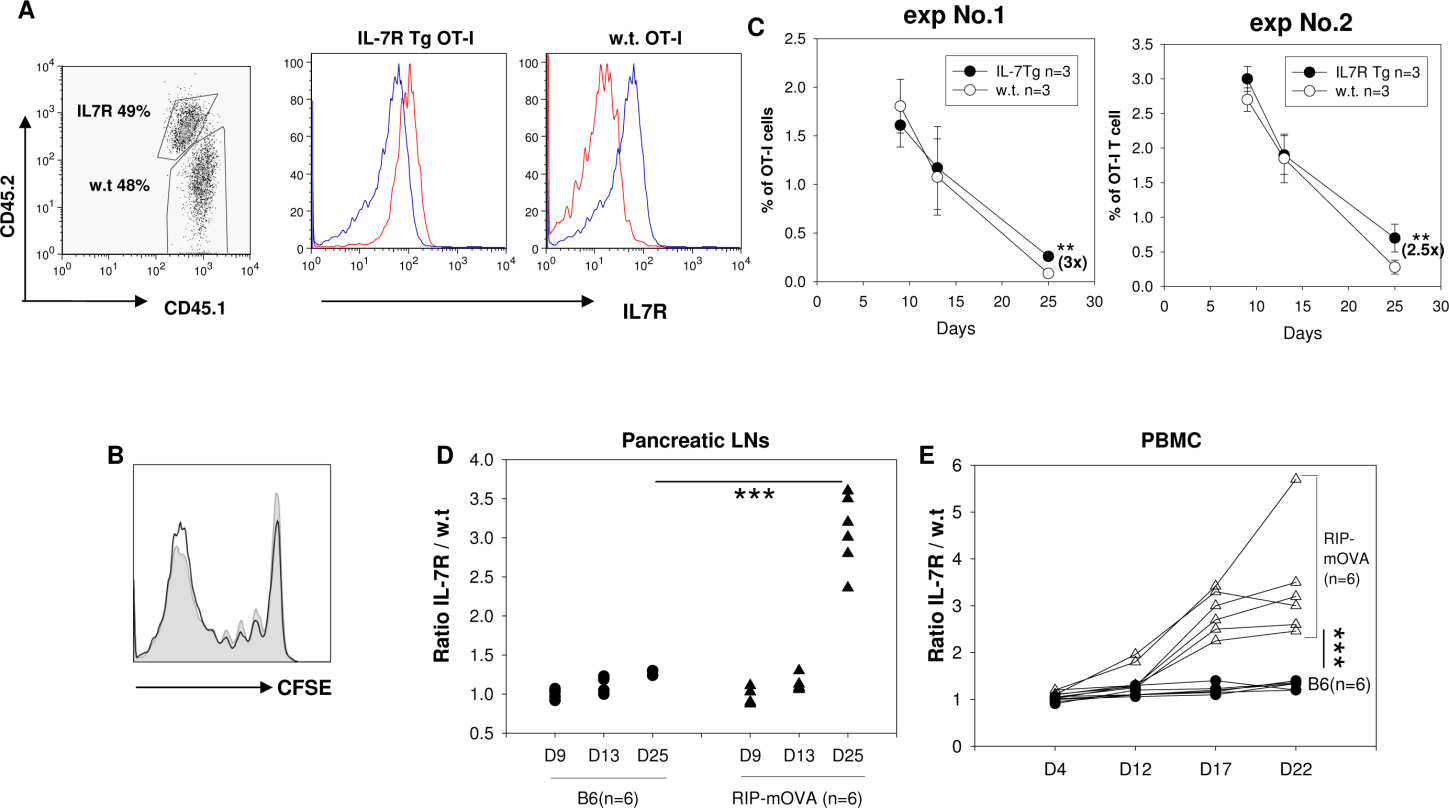
Over-expression of IL-7R promotes OT-I T cell survival in RIP-mOVA mice through cell intrinsic mechanism. 0.5 x 10^6^ purified CD45.1+/CD45.2+ IL-7R OT-I T cells were mixed with 0.5 x 10^6^ purified CD45.1+ w.t. OT-I T cells, labeled with CFSE, and transferred into RIP-mOVA mice (n = 3). **A:** At day 7 after transfer, total cells were recovered from the pancreatic LNs. IL-7R Tg OT-I cells were distinguished from w.t. OT-I T cells by their congenic markers. The expression of IL-7R on the transferred cells (red) was compared with that of endogenous T cells (blue). As expected, IL-7R on IL-7R Tg OT-I T cells maintained high levels of expression. The result is representative of at least 5 experiments. **B**: Proliferation of transferred OT-I T cells in **A** was measured by flow cytometric analysis of CFSE dilution (gray filled–w.t. OT-I T cells, black solid line–IL7R Tg OT-I T cells). The results are representative of two experiments (n = 3 in each experiment). **C:** Purified T cells were mixed and co-transferred into RIP-mOVA mice as in **A**. The percentages of IL-7R Tg and w.t. OT-I T cells recovered from the pancreatic draining LNs at day 9, 13, and 25 after the transfer (n = 3 at each time point). The results from two independent experiments are shown. ** p<0.001, student t test. **D-E**: The ratios between IL-7R Tg and w.t. OT-I T cells in the pancreatic draining LNs (**D**) and PBMC (**E**) are shown. The results are pooled from the two independent experiments as depicted in **C**.

The increased recovery of IL-7R Tg OT-I cells at day 7 can be caused either by extrinsic factors, such as an increased release of OVA antigen from the pancreas, or by an intrinsic property of the IL-7R Tg cells. To differentiate these two possibilities, we followed the fate of IL-7R Tg and w.t. OT-I T cells in the same recipient. We mixed CD45.1+CD45.2+ IL-7R Tg OT-I T cells and CD45.1+ w.t OT-I T cells at a 1:1 ratio, labeled them with CFSE, and transferred them into RIP-mOVA mice. We evaluated the CFSE dilution of OT-I T cells at day 7 after the transfer. Despite the sustained IL-7R expression by IL-7RTg OT-I T cells in the pancreatic draining LNs (**Fig 4A**), they showed a similar decrease in CFSE as w.t. OT-I T cells (**Fig 4B**), suggesting the increased recovery of IL-7R Tg OT-I T cells at day 7 (**Fig 3C)** was not caused by the intrinsic ability of IL-7R to induce proliferation.

Deletion of self-reactive T cells is an important mechanism of tolerization [33]. Over-expression of IL-7R may protect activated OT-I T cells from deletion. However, we found that between day 1 and day 25 after transfer, both IL-7R Tg and wt. OT-I T cells decreased substantially in number, indicating over-expression of IL-7R did not overcome self antigen mediated deletion (**Fig 4C**). However, the number of surviving IL-7R Tg OT-I cells at day 25 was 3-fold higher than w.t OT-I cells (**Fig 4C and 4D.** In **Fig 4C,** exp No.1: IL-7R Tg 0.26+/-0.036 vs. w.t. 0.08+/-0.01 p< 0.01 n = 3, exp No.2: IL-7R Tg 0.7+/-0.2 vs. w.t. 0.28+/-0.1 p< 0.01 n = 3). When monitored using peripheral blood, the divergence between IL-7RTg OT-I and w.t OT-I T cell could be observed at day 17 post transfer (**Fig 4E**). Importantly, the improved survival of IL-7R Tg OT-I T cells was driven by antigen, as IL-7R Tg and w.t OT-I T cells retained their initial 1:1 input ratio in antigen-free B6 mice even at day 25 after the transfer.

### Forced expression of IL-7R enhances CD8 T cell cytotoxicity in the target tissue in RIP-mOVA mice

**Fig 5:**
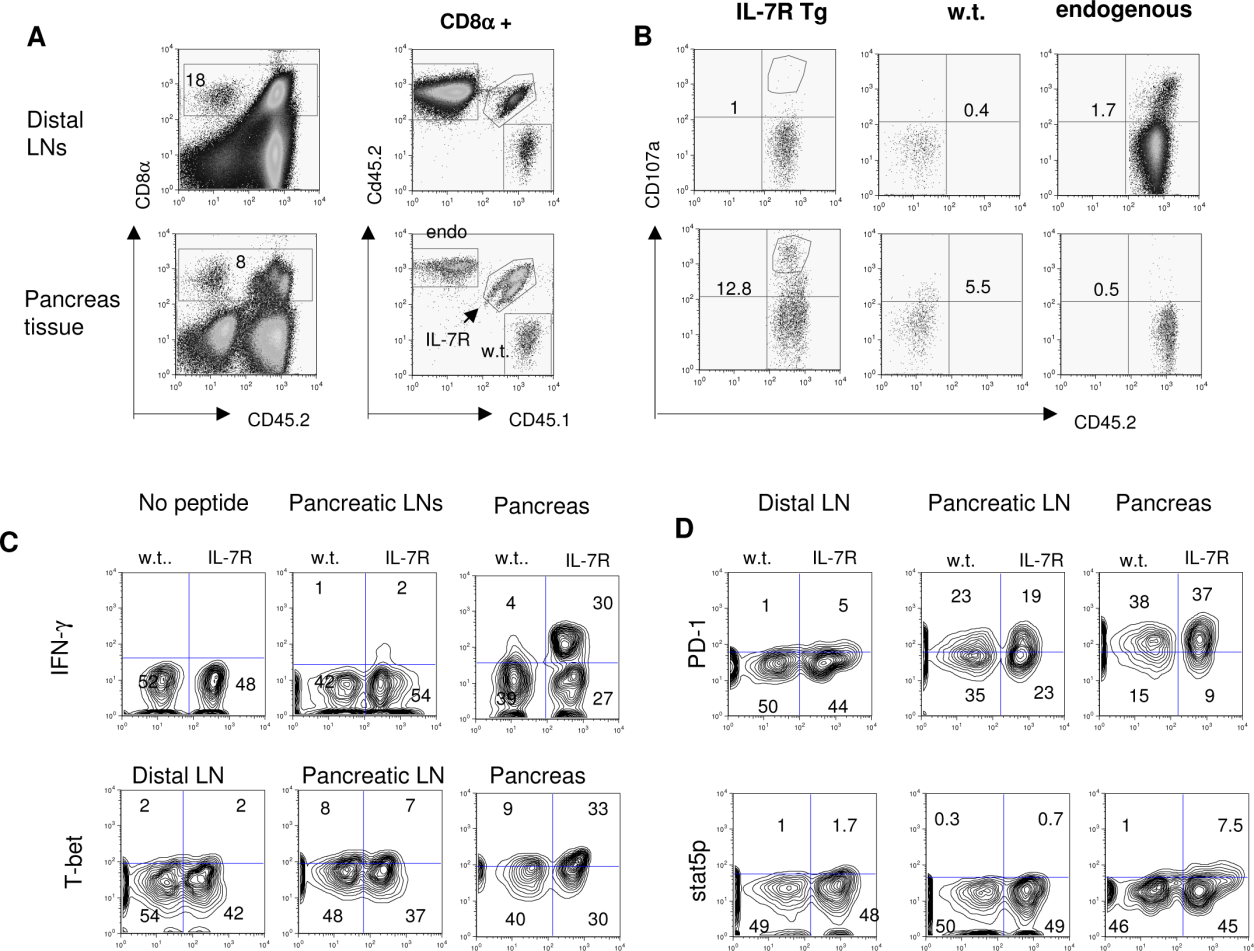
Over-expression of IL-7R enhances OT-I T cell effector function in the pancreas. **A and B:** 0.5 x 10^6^ purified CD45.1+ w.t. OT-I T cells were mixed with 0.5 x 10^6^ purified CD45.1+/CD45.2+ IL-7R Tg OT-I T cells and transferred into RIP-mOVA mice (n = 5). At day 6 after the transfer, pancreata from the recipients were pooled and islets were separated. Infiltrating lymphocytes were isolated from the islets as described in Material and Methods. 5x10^4^ viable cells were recovered. Endogenous T cells (endo), w.t. and IL-7R Tg OT-I T cells were distinguished by their congenic markers (**A**) and their surface exposure of CD107a was determined in **B** by flow cytometry. Pooled T cells from distal LNs were used as an internal negative control. **C and D:** Intracellular expression of IFN-γ, T-bet, pSTAT5, and surface expression of PD-1 by OT-I T cells recovered from different sites (pooled from 5 mice in each experiment). In order to detect IFN-γ production, pooled lymphocytes were re-stimulated with OVA peptide for 5 hours before fixation. The results from **A-D** are representative of two experiments.

Since IL-7R Tg OT-I T cells showed only a modest improvement in long term survival, we investigated whether enhanced effector function could explain their significantly higher efficiency in inducing diabetes in RIP-mOVA mice. To this end, we co-transferred IL-7R Tg OT-I and w.t. OT-I T cells into RIP-mOVA mice. We isolated lymphocytes from the pancreata and LNs at day 7 after transfer (**Fig 5A**) and compared markers of CD8 T cell functionality. As shown in **Fig 5B**, IL-7R Tg OT-I T cells isolated from the pancreata showed increased externalization of CD107a, a marker associated with de-granulation. Moreover, they expressed higher IFN-γ and T-bet than w.t OT-I T cells from the same environment (**Fig 5C**). Interestingly, the effect of IL-7R Tg was only observed in the pancreata but not in the draining LNs, suggesting IL-7R regulated the cytotoxicity but not the priming of CD8 T cells. Although manipulating the IL-7/IL-7R pathway modulated the expression of PD-1 on CD4 T cells [13], we found that IL-7R Tg had no effect on PD-1 expression on OT-I T cells from either the pancreata or the draining LNs **(Fig 5D**).

**Fig 6:**
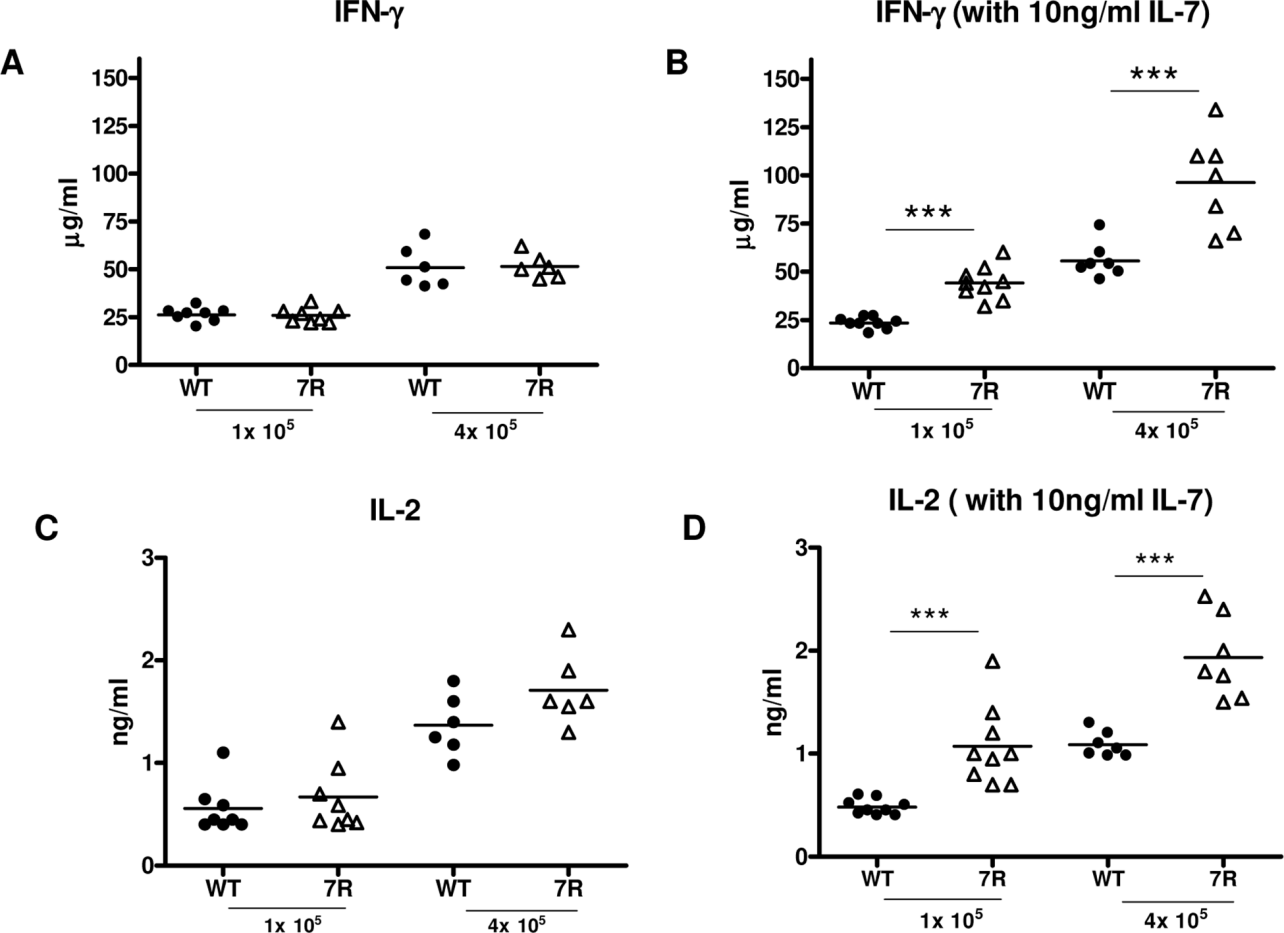
Forced expression of IL-7R increases IL-2 and IFN-γ production by OT-I T cells *in vitro*. 1 x 10^6^ purified w.t. or IL-7R Tg OT-I T cells were stimulated with different numbers of apoptotic thymocytes from actin-mOVA transgenic mice. In B and D, murine recombinant IL-7 (10ng/ml) was added to the culture. At day 3 after stimulation, the levels of IFN-γ (A, B) and IL-2 (C, D) in the supernatant were measured by ELISA. The results were pooled from two independent experiments.

To further test the effect of IL-7R Tg on CD8 T cell function, we stimulated OT-I cells with different numbers of apoptotic cells from actin-mOVA mice *in vitro* in the presence or absence of recombinant IL-7. As shown in **Fig 6,** in the presence of IL-7, IL-7R Tg OT-I cells produced 2–3 fold more IL-2 and IFN-γ than w.t. OT-I T cells.

### Forced expression of IL-7R acts in synergy with PDL-1 deficiency to further enhance cytotoxicity to self antigen

**Fig 7:**
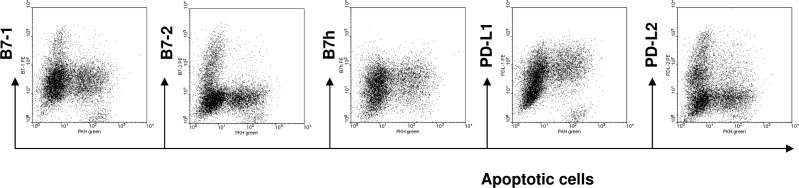
Immature DCs ingesting apoptotic cells expresses high levels of PD-L1. Bone marrow derived immature DCs were mixed with PKH green labeled apoptotic cells at a 1:5 ratio. At 12 hr after incubation, the expression of costimulatory and co-inhibitory molecules on CD11c+ PKH+ DCs was evaluated by flow cytometry. The results are representative of 5 experiments.

The tolerogenic ability of immature DCs can be attributed to their low expression of costimulatory molecules on their surface. To determine the contributions from co-inhibitory molecules such as PDL-1 and PDL-2, we evaluated their expression on immature DCs that have ingested apoptotic cells. We mixed PKH green labeled apoptotic cells with day 6 immature DCs at a 5:1 ratio, and analyzed the expression of costimulatory and co-inhibitory molecules on the CD11c+PKH+ cells. As shown in **Fig 7**, compared with PKH- immature DCs, PKH+ immature DC that contained apoptotic cells not only had lower expression of B7-1 and B7-2, they also expressed higher level of PDL-1 but not PDL-2 on their surface.

**Fig 8:**
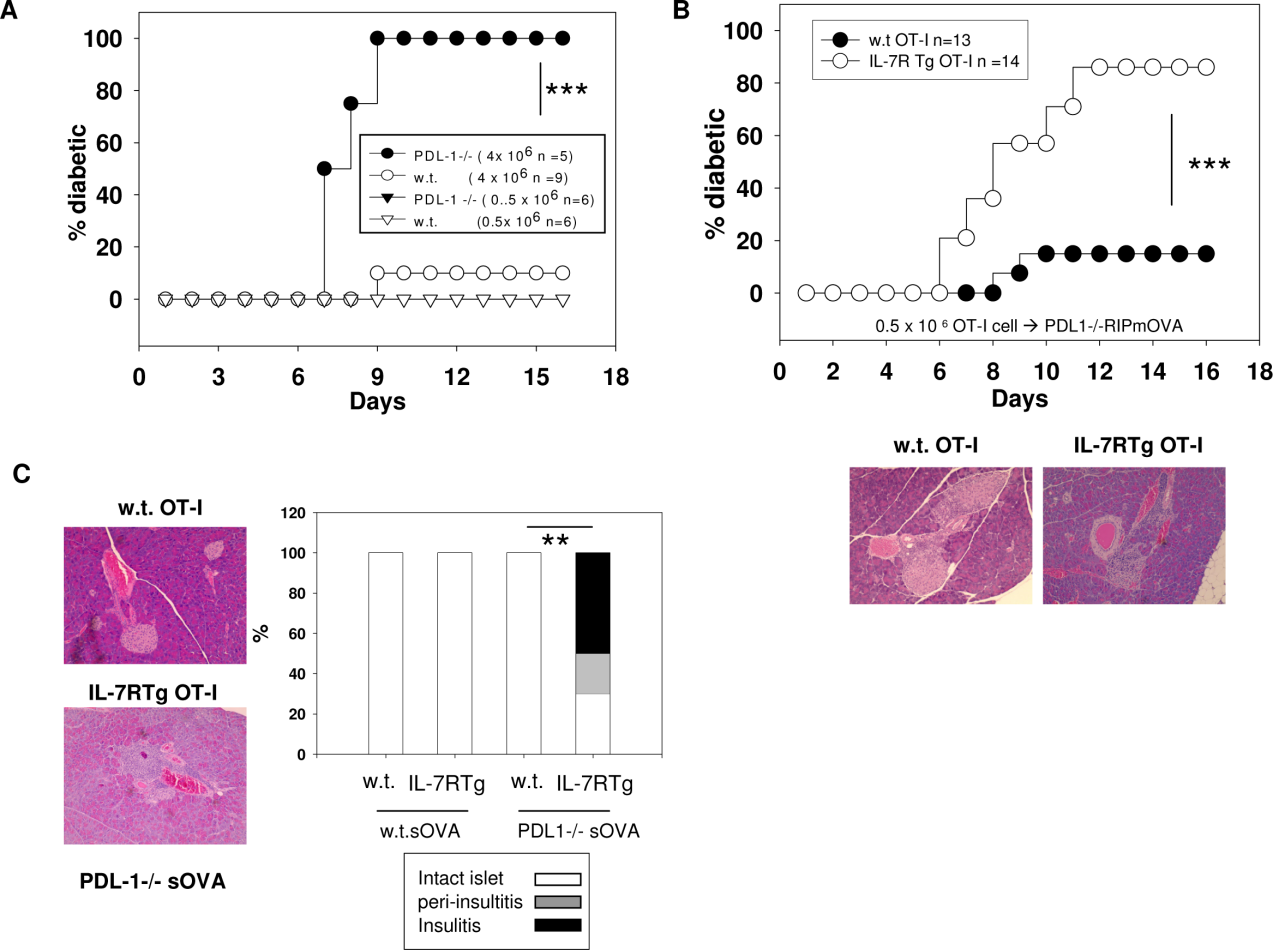
Forced expression of IL-7R and PDL-1 deficiency acts in synergy to promote cytotoxic response to self antigen. **A:** 4x 10^6^ or 0.5x 10^6^ w.t. OT-I T cells were injected into w.t. or PDL-1-/- RIP-mOVA mice (n = 5–9 in each group). The recipients were monitored for diabetes by urine analysis. *** p< 0.001 by log-rank test. **B:** 0.5 x 10^6^ w.t or IL-7R Tg OT-I T cells were transferred into PDL-1-/- RIP-mOVA mice (n = 13–14 in each group). The recipients were monitored for diabetes by urine analysis. *** p< 0.001 by log-rank test. Representative H&E staining of the recipients’ pancreata are shown. **C:** 5 x 10^6^ w.t. or IL-7R Tg OT-I T cells were transferred into w.t. or PDL1-/- RIP-sOVA mice (n = 5 in each group). Pancreata were collected from the recipients at day 14 after transfer. Representative H&E staining of the pancreata from the PDL1 -/- RIP-sOVA recipients are shown. Right: summary of insulitis (10 islets from each pancreas were scored as **Fig 3B**). ** p<0.01 by Mann-Whitney U test.

We next tested whether IL-7R Tg would further boost CD8 T cell cytotoxicity in PDL-1 deficient mice. We first crossed PDL-1-/- with RIP-mOVA mice. Consistent with previous studies [34], after injecting 4 x10^6^ w.t. OT-I cells, 5/ 5 PDL-1-/- and 1/9 w.t RIP-mOVA recipients developed diabetes. However, the effect of PDL-1 deficiency depended on the initial input number of OT-I T cells, as 0.5 x 10^6^ w.t. OT-I T cells failed to induce diabetes even in PDL-1-/- RIP-mOVA mice (**Fig 8A**). Forced expression of IL-7RTg markedly enhanced the function of OT-I cells in PDL-1 deficient mice. As shown in **Fig 8B**, 0.5 x 10^6^ IL-7Tg OT-I cells were able to induce diabetes in 80% of the PDL-1-/-RIP-mOVA mice and extensive infiltration of lymphocytes could be observed in the islets of recipients. On the contrary, the same number of IL-7R Tg OT-I cells failed to induce diabetes in w.t. mOVA mice (**Fig 3A**).

Self antigens with weak immunogenicity can evade detection by self reactive CD8 T cells through ignorance. The form of self antigen determines their immunogenicity. In the RIP-sOVA model, soluble OVA expressed by β cells is much less effective in priming OT-I T cells than membrane bound OVA [31]. To determine whether the combination of IL-7R Tg and PDL1 deficiency would overcome the weak immunogenicity of soluble OVA, we injected 5 x 10^6^ w.t. or IL-7R Tg OT-I T cells into w.t. or PDL1-/-RIP-sOVA mice. Although none of the recipients became overtly diabetic, 3/5 PDL1-/-mice injected with IL-7R Tg OT-I T cells showed extensive insulitis, whereas all other recipients remained free of leukocyte infiltration (**Fig 8C**).

### Forced expression of IL-7R does not reverse T cell anergy

**Fig 9:**
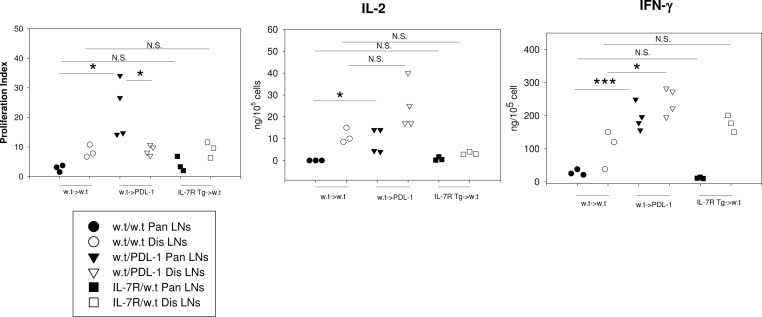
Forced expression of IL-7R does not reverse T cell anergy. 5x 10^6^ w.t. OT-I T cells were transferred into w.t. or PDL-1-/- RIP-mOVA mice (n = 3–4 in each group). In a separate group, 5x10^6^ IL-7 Tg OT-I T cells were transferred into w.t. RIP-mOVA mice (n = 3). At day 7 after transfer, total cells (>90% viable) from pancreatic draining LNs (primed-closed symbol) and distal draining LNs (naïve-open symbol) were harvested and re-stimulated with OVA peptide for 3 days *in vitro*. T cell proliferation was assayed by adding H^3^ thymidine to the culture during the final 14 hours. The levels of IL-2 and IFN-γ in the supernatant were assessed by ELISA. The proliferation index was calculated as: H^3^ counts of stimulated T cells/ H^3^ counts of un-stimulated T cells. N.S. = not significant.

Tolerized CD8 T cells are resistant to restimulation. In RIP-mOVA mice, primed OT-I T cells from the pancreatic draining LNs neither proliferated nor produced cytokines upon peptide stimulation *in vitro*. In accordance with the reversal of CD8 T cell anergy in PD-1 deficient mice [21], primed OT-I T cells from PDL-1-/- RIP-mOVA mice regained their abilities to produce IL-2 and IFN-γ and proliferated more vigorously *in vitro* than unprimed cells from distal LNs. On the contrary, despite its ability to enhance cytotoxicity and to improve survival, IL-7R Tg did not reverse the anergic state of primed CD8 T cells (**Fig 9**).

## Discussion

During chronic viral infection and tolerization of self reactive T cells, continuous engagement between antigens and TCRs down–regulates the IL-7R expression on antigen specific T cells. The source of antigenic peptide is likely to be generated by immature DCs that have engulfed apoptotic cells *in vivo*. In addition to their low expression of costimulatory molecules and their lack of cytokine expression, immature DCs have a longer life span than mature DCs *in vivo* [2]. Whereas the short life span of mature DCs allows effector T cells to quickly recover their IL-7R expression, the continuous presentation by immature DCs can impair the function of self reactive T cells by depriving them of IL-7 signaling.

In this paper, forced expression of IL-7R only partially reversed the fate of self reactive T cells. TCR mediated deletion remained largely intact. IL-7R only provided a survival benefit at a late stage (> 17 days). Indeed, the expression of Bcl-2 remained depressed in activated IL-7R Tg OT-I T cells in the pancreas. The limited amount IL-7 *in vivo* may not be sufficient to counter the deletional effect of TCR signals. Constraints on available IL-7 may also explain why the levels of integrins on IL-7R Tg OT-I T cells are similar to w.t. OT-I T cells, though recombinant IL-7 had been shown to modify integrin functions to promote tissue migration of CD8 T cells in a model of sepsis [35]. Alternatively, over-production of IFN-γ by IL-7R Tg OT-I cells may also promote their own apoptosis [11].

Interestingly, most of the effects of IL-7R in our model were confined to the target tissue, suggesting IL-7R may boost the effector function but not the priming of autoreactive CD8 T cells. Since the majority of OVA antigen in RIP-mOVA mice is expressed in the pancreas not LNs, it is very possible that the enhancing effect of IL-7R requires simultaneous engagement of a large number of TCRs on the surface.

T cell tolerization *in vivo* can be mediated by co-inhibitory molecules such as PDL-1. It is interesting that uptake of apoptotic cells in immature DC culture is exclusively limited to the PDL-1 high population. Sorted PDL-1 high immature DCs were much more efficient than PDL-1 low immature DCs in phagocytosis. Although the GM-CSF derived immature DCs in our study are heterogeneous [36] and their physiological counter-parts are not clearly defined *in vivo*, a recent proteomics study suggests their similarity to inflammatory macrophages generated during peritonitis [37]. Their phenotype also resembles some of the immature myeloid suppressor cells in tumor-bearing mice [38]. Moreover, a few subsets of myeloid cells specialized in phagocytosis also express high levels of PDL-1 [17]. Whether the high expression of PDL-1 in subsets of BMDCs is mechanistically linked to their phagocytic ability needs further clarification.

The PDL1-PD1 interaction suppresses T cell effector function rather than their priming [39]. PDL-1 deficiency and PDL-1 blockade can reverse the anergic phenotype of autoreactive T cells [16]. Recent trials of anti-PDL1 antibody in immuno-therapy demonstrated restored function of self reactive CD8 T cells in cancer patients. The function of IL-7R Tg revealed in this study has the potential to synergize with the PDL-1 blockade to break CD8 T cells tolerization as well as their ignorance to self antigens. Forced-expression of IL-7R may therefore represent another strategy to overcome the T cell suppression imposed by continuous presentation of tumor antigen by immature myeloid cells.
